# Patient Phenotypes Undergoing Tricuspid Transcatheter Edge-to-Edge Repair: Finding the Optimal Candidate

**DOI:** 10.3390/jcdd12080293

**Published:** 2025-07-31

**Authors:** Kyriakos Dimitriadis, Nikolaos Pyrpyris, Eirini Beneki, Panagiotis Theofilis, Konstantinos Aznaouridis, Aggelos Papanikolaou, Alexios Antonopoulos, Christina Chrysohoou, Konstantina Aggeli, Konstantinos Tsioufis

**Affiliations:** 1 First Department of Cardiology, School of Medicine, National and Kapodistrian University of Athens, Hippokration General Hospital, 115 27 Athens, Greece; npyrpyris@gmail.com (N.P.); e.beneki@hotmail.com (E.B.); panos.theofilis@hotmail.com (P.T.); conazna@yahoo.com (K.A.); agepap25@otenet.gr (A.P.); antonopoulosal@yahoo.gr (A.A.); chrysohoou@usa.net (C.C.); dina.aggeli@gmail.com (K.A.); ktsioufis@gmail.com (K.T.); 2 Department of Cardiology, Lausanne University Hospital and University of Lausanne, 1005 Lausanne, Switzerland

**Keywords:** tricuspid regurgitation, transcatheter edge-to-edge repair, chronic kidney disease, atrial fibrillation, cardiac electronic implantable device

## Abstract

Tricuspid regurgitation (TR) is a well-recognized factor contributing to adverse outcomes and mortality. Recent developments in transcatheter valve repair techniques, with the emergence of tricuspid transcatheter edge-to-edge repair (TEER) devices, have altered the treatment algorithm of TR and now offer a safe and feasible alternative for the effective management of the disease and an improvement in patient symptoms. Evidence from large studies and registries showcases the benefit of tricuspid interventions in terms of heart failure hospitalization and quality of life; however, most studies do not report a significant benefit in terms of hard outcomes. Even though longer-term follow-up may be needed to identify such differences, it is important to also identify distinct patient phenotypes that would benefit the most from such interventions, moving from pure anatomical criteria to an overall assessment of the patient’s clinical status. Therefore, the aim of this review is to provide updates on potential moderators of the effect of tricuspid TEER, focusing on novel anatomical criteria, right cardiac function, and renal physiology, in order to guide patient selection and provide an insightful discussion on the optimal patient phenotype for future trial design.

## 1. Introduction

Tricuspid regurgitation (TR) has been increasingly recognized in recent years as one major cause of morbidity and mortality. It is estimated that approximately 4% of elderly individuals suffer from this pathology, while the prevalence further increases with a more advanced age [1]. However, despite the significant proportion of patients, TR remains underdiagnosed and undertreated, resulting in increase of adverse events. Notably, isolated TR is related to suboptimal survival at both 1- and 5-year (86 and 54%) follow-ups, respectively [2]. Despite this poor survival, only 4–5% of patients with moderate/severe TR are reportedly being treated in large nationwide registries [3]. Mechanistically, TR can be categorized as primary (5%) or secondary (95%). The most common etiologies of secondary TR are left-sided heart disease, pulmonary hypertension, and right ventricular dysfunction [4]. Nevertheless, even though TR secondary to left-sided disease accounts for the majority of cases (55%), studies provide novel information regarding isolated TR, defined as the presence of TR in the absence of valvular disease, pulmonary hypertension, or left heart disease, which, despite being less common, is an increasingly recognized target for early intervention [5].

Until the emergence of transcatheter therapies, TR has been managed by either conservative, medical treatment or surgery. However, no specific pharmacotherapy has been proven to reduce mortality events in this pathology, while the high surgical risk of these patients has resulted in avoiding such operations due to increased adverse events. Importantly, even the most recent studies report an increased 30-day mortality after tricuspid valve surgery, reaching 15% [6], which is further increased in the presence of comorbidities such as right ventricular (RV) dysfunction [7,8,9]. However, it should be noted that these suboptimal results were related to the delayed referral of patients for surgery, as prompt interventions have been associated with enhanced post-procedural results. This has been noted by studies using the novel risk stratification tool TRI-SCORE, showing that intervening in patients at a lower disease stage, as indicated by a lower baseline TRI-SCORE, is a key factor for optimizing patient outcomes [10,11,12]. Recently, the introduction of transcatheter edge-to-edge repair (TEER) has further increased the treatment options for such patients. However, to date, the use of such devices has not been proven to have benefits in terms of mortality, leading to questions regarding the effect of the TR mechanism, tricuspid valve morphology, and patient comorbidities in patient outcomes. Therefore, the aim of this review is to provide an overview of large trials and real-world registries documenting the safety of TEER for TR (T-TEER), discuss recent findings of TEER studies for different patient phenotypes, and highlight potential predictors of success that may be used in clinical practice and research settings to evaluate the true benefits of TEER in terms of patient survival.

## 2. Major Trials in T-TEER

The use of T-TEER for patients with TR started in 2015, with the use of non-dedicated mitral TEER devices (MitraClip). Specifically, Nickenig et al. [13] enrolled 64 patients with severe TR who were unsuitable for surgery and were under optimal medical treatment, in whom a MitraClip device was implanted for compassionate use. The grade of TR was severe or massive in the majority of patients (88%), and most enrolled individuals had secondary TR (88%). The procedural success of the MitraClip implantation was 97%, with no major periprocedural complications; however, three in-hospital deaths were reported. TR severity was reduced by at least one grade in 91% of patients, while significant improvements were also noted in New York Heart Association (NYHA) class and six-minute walking test distance. Positive results with the use of the non-dedicated MitraClip device were also noted at the 1-year follow-up in the TriValve registry [14], showing a sustained reduction in TR grade (TR ≤2+ in 72% of patients) as well as a functional status improvement (NYHA class ≤II in 69% of patients). Of note, this study also provided early markers of procedural failure and adverse events, which included effective regurgitant orifice area, tricuspid coaptation gap, tricuspid tenting area, the absence of central or anteroseptal TR jet location and procedural failure, worsening kidney function, and the absence of sinus rhythm, respectively.

Further studies were designed to evaluate the use of the dedicated T-TEER systems, proving their safety and efficacy in significantly improving TR as well as providing symptom relief and an improvement in quality of life at 1-year follow-up (Table 1) [15,16,17,18]. The most long-term follow-up to date was provided by Nickenig et al. [19], who recently reported the 3-year results of the TRILUMINATE single-arm study. The study evaluated 54 patients from the total original cohort who underwent T-TEER with a 3-year follow-up. A reduction to moderate or lower levels of TR at 3 years was observed in 79% of subjects (*p* < 0.0001 from the baseline), while the change in TR grade between 1 year and 3 years was not significant (*p* = 0.912), confirming the sustained benefit of the therapy. Moreover, a TR reduction of at least one grade was achieved in 92% of subjects, while the proportion of subjects classified as NYHA class III/IV decreased from 76% at baseline to 19% at 3 years (*p* < 0.0001 from the baseline), with no difference between the 1- and 3-year follow-ups (*p* = 0.125). In regard to clinical events, the investigators note that subjects achieving a TR reduction to moderate or lower levels at 30 days were less likely to experience HF hospitalization or death at 3 years compared to those with residual severe TR (30.7% vs. 50.7%; HR: 0.48; *p* = 0.026). Finally, the investigators showed that 44% of patients had further regression of TR at 3 years, compared to 1 year, while 19% had progression, which merits further investigation, considering the natural history of the disease [20].

Recently, two significant randomized trials compared the use of T-TEER versus medical therapy in patients with TR. The TRILUMINATE-Pivotal trial [21] randomized a total of 572 patients with severe, symptomatic TR to either T-TEER and medical therapy or medical therapy alone. At the 1-year follow-up, the win ratio analysis for the primary endpoint (freedom from all-cause death, tricuspid valve surgery, HF hospitalization, and improvement in KCCQ score) favored the TEER group (win ratio, 1.48; 95% CI, 1.06 to 2.13; *p* = 0.02). However, all-cause mortality or HF hospitalization events were not found to be significantly different between groups, despite substantial improvements in quality of life. Regarding the extent of TR improvement, 87.0% of patients in the T-TEER group and 4.8% in the control group had no greater than moderate TR at 30 days (*p* < 0.001), with these results being sustained at the 1-year and 2-year follow-up (84%) [22]. Importantly, at 2 years, the annualized rate of HF hospitalizations was significantly lower with T-TEER compared to medical treatment, resulting in a 28% relative risk reduction in the device group (0.19 events per patient-years versus 0.26 events per patient-years; *p* = 0.02). Similar results were also reported in the Tri.Fr study [23], including 300 patients undergoing T-TEER or conservative therapy, with the composite clinical endpoint (change in NYHA, patient global assessment, or the occurrence of MACE) being in favor of T-TEER compared to medical therapy alone (improvements of 74.1 vs. 40.6%, respectively).

Along with the use of the TriClip system, another similar system (Pascal) has been designed for T-TEER and is currently being tested in clinical trials. Early studies [24] comparing the Pascal with the MitraClip system for T-TEER showed similar procedural success (92%) as well as device complication rates (10.4 vs. 15.3%, *p* = 0.59), with both groups experiencing significant functional (NYHA class III/IV 36% and 25%, respectively, both from the baseline; *p* < 0.001) and exercise improvements (six-minute walking distance improved from 216 ± 119 m at the baseline to 291 ± 121 m at the 30-day follow-up in the MitraClip group and from 213 ± 115 m to 290 ± 122 m in the PASCAL group; both *p* < 0.01). More recently, the CLASP TR early feasibility study [18] also reported favorable results with the use of the Pascal Ace system. More specifically, they showed a significant reduction in TR severity (*p* < 0.001), with 86% of patients achieving moderate or lower levels of TR and 100% having at least one TR grade reduction at the 1-year follow-up. Moreover, significant improvements were also noted regarding the NYHA class (92% NYHA class I/II; *p* < 0.001 from baseline), as well as in six-minute walk distance (+94 m; *p* = 0.014 from the baseline) and KCCQ score (+18 points; *p* < 0.001 from the baseline). Finally, the freedom from all-cause mortality and HF hospitalization at the time of follow-up were 87.9% and 78.5%, respectively.

Considering the diverse anatomies of patients with TR presenting in T-TEER and the absence of complex phenotypes from randomized settings, a recent sub-analysis of the TRILUMINATE single-arm study aimed to provide an overview of the safety and efficacy of T-TEER in such patients [25]. The sub-analysis included 100 patients with massive or torrential TR (98%), with 44% having prior left-sided valve interventions, 63% having four or more segmental tricuspid leaflet morphologies, 35% having CIED-related TR, and an average coaptation gap of 7.4 ± 2.7 mm. At the 1-year follow-up, 81% of subjects had moderate or lower levels of TR, showing the efficacy of T-TEER even in adverse anatomies. No major adverse events or deaths occurred within 30 days post-procedurally, while the 1-year all-cause mortality and HF hospitalization rates were 15% and 24%, respectively. Similar to previous studies, significant improvements in NYHA and KCCQ overall scores were shown and maintained to the 1-year follow-up.

TR is a heterogenous pathology, with several anatomic and pathophysiologic insights that may affect the efficacy of T-TEER and its ability to provide substantial clinical benefit. Therefore, along with established anatomic criteria for optimal patient selection, novel investigations have evaluated the safety and efficacy of T-TEER in distinct patient phenotypes, which could assist decision making, while increasing the evidence of and indications for offering T-TEER in clinical practice.

## 3. Echocardiographic and Anatomic Selection of T-TEER

Before establishing specific phenotypes of patients benefiting from T-TEER, available investigations have established anatomic criteria for the suitability or futility of T-TEER, in order to guide decision making regarding the intervention. In overview, Praz et al. have presented anatomic criteria based on which patients evaluated for tricuspid interventions are classified as favorable, feasible, and unfavorable for T-TEER, with the imaging of the tricuspid valve having an incremental role in this classification. Patients favorable for T-TEER are, therefore, individuals with a small septolateral gap (≤7 mm), anteroseptal jet location, tethering height ≤1 cm, confined prolapse, and flail and trileaflet valve morphologies [26]. Other morphologies, such as a large septolateral gap (>8.5 mm), dense chordae, leaflet thickening or shortening, the involvement of leads, or poor echocardiographic visualization of the leaflets, were considered unfavorable T-TEER anatomies, which would potentially benefit more from valve replacement [27]. Therefore, careful pre-procedural assessment employing multimodality imaging is crucial to understand the anatomy and mechanism of TR in each patient and decide whether T-TEER would be feasible, based on the established criteria.

Initial assessment of patients presenting with TR is commonly performed via ultrasound, including both transthoracic (TTE) and transesophageal (TEE) echocardiography, which are used to evaluate tricuspid valve morphology, TR severity and the extent of right heart remodeling [28]. TTE is the first-line method recommended to identify and evaluate patients with TR, as it can provide significant information regarding the tricuspid valve disease, as well as other notable abnormalities in cardiac or valvular function that could alter the patient selection process. TTE views commonly used during this index assessment are the parasternal long-axis and short-axis views, the apical four-chamber view, and subcostal view, which allows for the identification of tricuspid valve leaflets, even though it remains technically difficult and has disputable results due to the varying degrees of transducer angulation and anatomical variations of the valve [29], as well as baseline severity evaluations via quantitative and qualitative methods [30].

Even though TTE is a sufficient examination for the basic assessment of valve anatomy and disease mechanism, the most commonly used modality for assessing transcatheter intervention eligibility, as well as guidance, is TEE. Even though imaging the tricuspid valve with TEE is more challenging than the mitral valve, due to its anterior position, it overcomes the limitation of poor posteroseptal and anteroposterior commissure visualization posed by TTE [31]. Commonly used views are the mid-esophageal, distal-esophageal, and transgastric views. In particular, the mid-esophageal window can be used to evaluate the valve, right ventricle, and their relation to the surrounding anatomic structures; the distal esophageal window can be used to optimize leaflet visualization; and the transgastric level can be used for 2D/3D en face imaging of the valve and chordae as well as the subvalvular mechanisms, the optimization of TR jet color, and spectral evaluations [32]. Simultaneous 2D-TEE biplane imaging, derived from a 3D-TEE matrix probe, is also recommended and is particularly useful for optimal leaflet morphology and function evaluation in all the aforementioned views [33]. Along with providing information on the anatomical criteria for T-TEER suitability during pre-procedural assessment, such as coaptation gap, jet location, leaflet morphology, potential lead interaction with the valve, and echocardiographic window, TEE is also used in assistance to fluoroscopy to guide clip insertion and apposition. Specifically, TEE is used to guide the insertion and apposition of the implanted device throughout the procedure, with the transgastric views commonly used for leaflet imaging and device orientation/positioning and the mid-esophageal views used for assessing leaflet insertion via 3D live reconstruction [34]. Finally, TEE can be used post-procedurally, to document procedural success and the need for further devices to eliminate residual TR, especially considering the adverse impact of residual TR in terms of patient outcomes [35].

Beyond the traditional parameters that were used in the major trials to include patients for T-TEER, novel TEE-based measurements could also increase the identification of optimal patients for T-TEER, via linking pre-procedural factors with the extent of post-procedural TR. One such parameter is the leaflet-to-annulus index (LAI), which represents the mismatch between leaflet length and annulus dilation and can indicate suboptimal leaflet coaptation and thus TR reduction. In this context, Tanaka et al. [36] showed that patients with residual TR ≥3+ have a significantly lower LAI compared to those with residual TR <3+ (1.04 ± 0.10 vs. 1.13 ± 0.09; *p* = 0.001), while LAI was associated in a multivariate analysis with the presence of significant residual TR ≥3+, independently of baseline TR severity or coaptation gap. However, this finding is not consistent in all patients, as a study by Pizzino et al. [37] indicated that inefficient TR reduction is associated with lower septal-lateral LAI values (which were assessed by Tanaka et al. [36]), but higher antero-lateral LAI values. According to the investigators, this discrepancy could be attributed to the higher complexity of patients included in their cohort, compared to the study by Tanaka, as indicated by the higher need to grasp the posterior leaflet (60 vs. 20%) and the need for more than one device in 72% of cases. Therefore, the use of novel indices and comprehensive TEE assessment beyond the typical anatomic criteria, even in highly complex patients, can improve T-TEER decision making and help select patients with higher odds of procedural success, with future studies needed in order to incorporate such markers in clinical practice.

## 4. T-TEER and Chronic Kidney Disease

Chronic kidney disease (CKD) is known to be associated with cardiovascular disease; in particular, almost half of patients with CKD stage 4 or 5 also have cardiovascular disease, while patients with advanced stage CKD have double the risk for cardiovascular mortality compared to normal individuals with sustained renal function [38,39]. Patients with TR have an increased prevalence of renal dysfunction, with studies estimating the percent of patients with both conditions at 45–50% [40]. Pathophysiologically, significant TR is related to worsening renal function, as a result of the associated volume overload and RV dysfunction. However, several other mechanisms may contribute to renal dysfunction, as the presence of RV dysfunction may lead tο decreased left ventricular (LV) cardiac output due to a reduction in RV cardiac output and therefore systemic hypoperfusion, while RV congestion secondary to RV remodeling can result in increased ventricular diastolic interdependence and therefore decreased LV filling [41]. The presence of RV dysfunction is key for this pathophysiologic pathway, as studies have shown that only the combination of significant TR and RV dysfunction, but not either independently, is associated with renal function deterioration [42].

As expected, the presence of renal dysfunction in TR patients is related with adverse cardiovascular outcomes. Butcher et al. [41] showed in a study of 1234 individuals with TR that a worse stage of renal function is significantly associated with worse survival at 1-year (85% vs. 87% vs. 68% vs. 58%) and 5-year (72% vs. 64% vs. 39% vs. 19%) follow-ups for stages 1, 2, 3, and 4–5, respectively. Notably, along with the stepwise association of CKD progression and TR grade with all-cause mortality, studies have shown that CKD progression and TR grade 1 have a comparable all-cause mortality rate with greater TR severity grades without CKD progression. Moreover, for patients with TR grade 1 alone, the risk for all-cause mortality doubles with worsening renal function during follow-up (OR 2.49 (95% CI 1.38–4.47), *p* = 0.002), highlighting the increased risk for adverse events added by the worsening of renal function even in less severe tricuspid valve disease [43].

The presence of CKD and worsening renal function also affects patients’ outcomes in the context of T-TEER. A recent analysis of the bRIGHT registry 1-year results, which evaluated the safety and efficacy of the TriClip device in a post-approval, real-world setting, showed that increased baseline serum creatinine (>1.2 mg/dL) was significantly associated with increased 1-year mortality rates (OR: 2.169; 95% CI: 1.494–3.147; *p* < 0.0001) [16]. A subsequent study by Jorde et al. [44], including 572 patients and comparing T-TEER with conservative treatment, showed that most subjects who died during the 12-month follow-up had moderate-to-severe renal impairment at baseline. Moreover, patients with moderate-to-severe CKD (eGFR < 45 mL/min/1.73 m^2^) at the baseline had an increased incidence of HF hospitalization (0.11 vs. 0.34 events/patient-year; *p* < 0.0001), regardless of treatment. Notably, when stratifying patients based on treatment method, the annualized HF hospitalization rate was numerically lower in the TEER arm (0.14 vs. 0.22 events/patient-year; *p* = 0.25) in patients with mild-to-moderate CKD, with no difference shown for moderate-to-severe renal impairment (0.34 vs. 0.39 events/patient-year; *p* = 0.61), showing that baseline CKD, especially that of increased severity, has a similar adverse effect on the outcomes of both T-TEER-treated and conservatively treated patients.

As T-TEER restores normal heart hemodynamics and relieves TR-associated congestion, it could have a beneficial effect on renal function and halt or improve renal decline (Table 2). One of the first studies directly evaluating the effect of T-TEER in renal function was performed by Karam et al. [45], which evaluated improvements in renal and liver function after TR interventions in 126 patients (59 isolated T-TEER; 67 combined mitral and tricuspid TEER). At the 6-month follow-up, renal function remained stable (50.7 ± 21.8 vs. 49.8 ± 21.9 mL/min/1.73 m^2^; *p* = 0.45), including that of patients with moderate-to-severe CKD (37.5 ± 11.9 vs. 40.1 ± 17.3 mL/min/1.73 m^2^; *p* = 0.39). Subsequently, the study by Jorde et al. [44] also evaluated changes in renal function in patients undergoing T-TEER. The investigators reported that, after an adjustment for the baseline eGFR, there was no difference in the change in eGFR value between the T-TEER and conservative arms (−0.10 ± 0.79 vs. −2.22 ± 0.82 mL/min/1.73 m^2^; *p* = 0.063) at the 12-month follow-up. However, a sub-analysis including only T-TEER patients with procedural success (moderate or lower levels of TR at discharge) indicated a statistically significant difference in the eGFR change between the two groups (+0.30 ± 0.85 vs. −2.27 ± 0.82 mL/min/1.73 m^2^; *p* = 0.03), even when examining only patients with baseline renal dysfunction (+3.55 ± 1.04 vs. +0.07 ± 1.10 mL/min/1.73 m^2^; *p* = 0.022), therefore indicating that the successful elimination of TR to a moderate or lower grade with the use of T-TEER may result in an improvement of renal function. Finally, along with successful TR reduction being a potential predictor of renal improvement, Felbel et al. [46] note that pre-interventional TAPSE, i.e., RV function, is also a predictor of renal function improvement in a multivariate regression model, while noting that an eGFR improvement of >9 mL/min could be associated with reduced 1-year HF hospitalization rates.

In light of the adverse effects that CKD and renal dysfunction in general have on TR patients, the available studies note that a successful intervention may be able to halt renal decline or even improve renal function. However, an improvement in clinical outcomes after T-TEER was only noted in patients with less than moderate renal disease. Therefore, future studies are needed to further investigate the effect of both T-TEER and TTVR on renal outcomes and renal biomarkers, as well as the association of any potential improvement in eGFR with a reduction in adverse events or enhanced quality of life. Importantly, identifying the optimal timing for offering T-TEER as well as predictors of post-procedural enhanced renal function in further investigations will aid patient selection and potentially lead to clinical improvement.

## 5. T-TEER and AFTR

Advancements in cardiovascular imaging and our understanding of the pathophysiology of secondary TR have allowed for the identification of a newly recognized subcategorization of functional TR in ventricular functional TR (VFTR) and atrial functional TR (AFTR). Specifically, even though secondary TR has been chronically attributed to RV dysfunction, leading to the dilatation of the tricuspid annulus and right atrial remodeling, recent investigations have identified a subset of patients that present with the latter but without RV dysfunction [47]. This entity, which was also described as isolated TR, can be present in 10–15% of clinically significant TR and is associated with the female sex, atrial fibrillation (AF), and HFpEF [47,48]. Of note, investigations with a mean follow-up of 13.3 years note that in patients with AF, about one in three patients will develop TR, which is associated with worse outcomes, compared to patients that do not develop TR [49]. In terms of pathophysiology, AFTR is a result of tricuspid annulus dilatation second to RA dilation and dysfunction, due to chronic remodeling related to the presence of atrial arrhythmias, thus resulting in an imbalance of the annulus diameter and the capability of the tricuspid leaflets to cover this increase [50]. It is known that AFTR, which is related to a lower comorbidity burden than VFTR, has a more favorable profile with enhanced survival at long-term follow-up, as well as fewer HF hospitalizations [51,52]. However, severe AFTR has been related to increased mortality and adverse events, compared to moderate or lower levels of AFTR [53,54], therefore necessitating prompt intervention in order to improve patient outcomes. In this context, several risk scores have been developed, encompassing clinical and right heart echocardiographic parameters, that could assist in timely patient selection for interventions [55].

Several studies have investigated the safety and efficacy of T-TEER for this phenotype (Table 3). Schlotter et al. [52] conducted one of the first analyses reporting results at a 1-month follow-up, which included 651 patients, out of which 213 underwent T-TEER. The TR reduction to moderate or lower levels was comparable between the AFTR and VFTR arms (86 and 81%, respectively; *p* < 0.001 from the baseline for both). Significant improvements from the baseline were also noted in functional outcomes, including the NYHA class and six-minute walking test distance. Importantly, AFTR was related to lower mortality and fewer HF hospitalization events compared to VFTR at one year (12 vs. 36%; *p* = 0.017). Similar results are also provided by Scheffler et al. [56], showing similar rates of TR reduction ≥ two degrees (92.6 vs. 96.3%; *p* = 0.34) or residual TR ≤ II (96.3 vs. 94.5%; *p* = 1.0), with a significant reduction in the composite of all-cause mortality and HF hospitalizations in the AFTR cohort (12.0 vs. 34.3%; *p* = 0.02), documenting similar procedural results and a trend towards better clinical outcomes in AFTR patients.

Larger and longer-term analyses are provided by two European T-TEER registries, namely the TriValve an EuroTR registries. Specifically, Russo et al. [57] reported a 1-year comparison of T-TEER in AFTR and VFTR patients from the TriValve registry, including a total of 298 patients (22% AFTR; 78% VFTR). Patients with AFTR were more commonly female, had lower rates of coronary artery disease and HF hospitalization for RV failure, a larger RA diameter, and a greater ejection fraction. As in previous studies, acute procedural success (80 vs. 83%; *p* = 0.56) and TR reduction to grade ≤ II (77 vs. 85%; *p* = 0.001 from the baseline for both) was not different between the two arms. However, survival was significantly increased at 1 year in the AFTR group (91 vs. 72%; *p* = 0.02). With the VFTR group, high-dose diuretics and acute procedural success were independent predictors of mortality. A larger analysis was provided by the EuroTR registry [58], encompassing a total of 641 patients (31% AFTR). Confirming previous studies, 50% of the AFTR patients were female and had increased rates of atrial fibrillation, decreased rates of other common comorbidities, better ventricular function, and a larger RA. In contrast to previous studies, the AFTR arm had a greater TR reduction at a grade ≤2+ (86.9 vs. 80.4% *p* = 0.005), as well as increased rates of NYHA class I/II at follow-up (62 vs. 54%; *p* = 0.033). At follow-up, the TR grade was similar between the two cohorts (TR ≤2+ in 74.8% and 70.6%, respectively; *p* = 0.353). Moreover, patients with AFTR had an increased rate of 2-year survival, which was associated with higher 2-year event-free HF hospitalization rates (66.3 vs. 47.5%; *p* < 0.001). Therefore, this study, besides including the largest cohort and documenting comparable safety and efficacy as VFTR, also potentially showed that T-TEER may achieve an even lower level of residual TR for this phenotype compared to VFTR.

The available studies, to date, showcase that T-TEER in patients with AFTR has similar feasibility and efficacy to VFTR, despite the inherent differences in the pathophysiology of the two conditions, which may be related to the more favorable clinical profile of AFTR patients and the absence of significant ventricular failure. Importantly, besides the significant reduction in TR and improvement of functional outcomes, these studies identified a significant benefit of T-TEER in HF hospitalizations in AFTR compared to VFTR. Therefore, timely interventions in this patient phenotype may lead to clinically important improvements, alongside symptomatic and functional benefits. Further studies exploring the pathophysiology of AFTR and the timing of the intervention, as well as comparing T-TEER outcomes in AFTR and VFTR, are necessary in order to identify the potential benefits of the procedure for each phenotype. Importantly, evaluating the use of T-TEER in comparison to other technologies, such as annuloplasty, is also needed, in order to assist optimal device selection for each distinct anatomy. Of note, early studies employing annuloplasty devices [59] report, similarly to T-TEER, a significant reduction in TR grade, which may be even more enhanced in VFTR patients, along with significantly fewer mortality events in AFTR patients. Understanding whether these observed benefits are linked to this phenotype itself or are attributed to an earlier stage of disease in those with AFTR is crucial in determining the optimal timing and candidates for T-TEER.

## 6. T-TEER and CIED

The presence of a cardiac implantable device (CIED) through the tricuspid valve is relatively common, as a lead can be found in approximately in 10% of patients with TR and 20–30% of patients with severe TR [60]. The presence of the lead may result in two distinct pathologies, i.e., lead-associated TR, in which the lead is not the causative mechanism of TR and its evolution depends on the usual risk factors identified for TR progression, and lead-related TR, in which the presence of the lead is the cause of the TR, which is associated with an increased rate of TR progression, with 20% of patients having an increase of at least one grade in a year [61]. Even though there are numerous management strategies, either focusing on the lead (lead extraction followed by other pacing strategies, such as leadless pacemakers) or the valve (surgery, transcatheter valve replacement, and T-TEER), each one has its associated risks and should be carefully proposed based on individualized criteria [62]. Namely, even though studies have shown that transcatheter tricuspid valve replacement can be performed in the presence of a lead, there is always a risk of lead entrapment, making it unable to be extracted, as well as lead failure. The available algorithms suggest lead extraction only in patients with lead-related TR and a favorable risk/benefit ratio for the procedure, while in all other instances, shared decision making for valve repair or replacement is required [63]. Even though the procedural complexity of T-TEER is increased in the presence of leads, due to the interaction of the lead with the valve and the device or shadowing during echocardiographic guidance, several studies provide insight on the safety and efficacy of T-TEER in this setting.

A few small studies have evaluated the safety and efficacy of T-TEER in the presence and absence of leads (Table 4). Braun et al. [64] evaluated a total of 41 patients undergoing T-TEER, out of which 13 had a pacemaker lead. Notably, the patients with leads were included if the presence of the lead was not considered the primary cause of TR. The baseline characteristics were not different between groups, and in respect to the procedure’s success, the rate of post-interventional TR grade ≤2+ was similar between arms (92 and 93%, respectively; *p* > 0.99). At the 30-day follow-up, the rates of TR grade ≤2+ remained the same as at the time of the post-procedural evaluation (*p* > 0.99). Moreover, significant improvements in NYHA class and six-minute walking test distance were observed in both cohorts. Similar results were reported by Lurz et al. [65] at the 30-day follow-up of a larger cohort, in regard to TR reduction and functional improvement. Moreover, they report that the result of T-TEER was more favorable (TR grade ≤1) in patients with commissural/central leads than in patients with leads across the leaflet (63 vs. 29%; *p* = 0.049). Finally, the investigators did not identify any major disruption of lead function, with the exception of three cases in which the pacing threshold increased.

Longer-term data are provided by Taramasso et al. [66], whose study included 470 patients with severe TR undergoing tricuspid interventions (including T-TEER in 79%), out of which 121 (25.7%) also had a CIED. In terms of baseline characteristics, patients with a CIED were more frequently NYHA class III/IV (95.9% vs. 92.3%; *p* = 0.02), had less severe TR, and had worse RV function. Of note, only a small number had TR induced by the lead (2.5%). In respect to procedural outcomes, no difference was noted in procedural success (78.6 vs. 80.0%; *p* = 0.74) or in-hospital mortality (3.7 vs. 2.9%; *p* = 0.70). Moreover, at the 30-day follow-up, a TR grade ≤2+ was achieved in similar rates in the two cohorts (73.7 vs. 70.8%; *p* = 0.6), with also no difference noted in NYHA class I/II (65.0 vs. 66.0%; *p* = 0.30). At the 1-year follow-up, no difference was noted in patient survival between the included patients (73.6 ± 5.0 vs. 80.7 ± 3.0%; *p* = 0.30). In contrast to the previous studies, Goebel et al. [67] analyzed 511 patients undergoing T-TEER, with a total of 110 having a lead, with 80.7% of these subjects having TR at least partially related to the lead. At 30 days, 71% of the patients with a CIED had a moderate or lower level of TR severity, along with significant improvements in functional outcomes and quality of life (*p* < 0.0001 from the baseline). Even though no direct comparison was made, the benefits in terms of functional and quality of life outcomes were of similar extent to those in the non-CIED cohort, as were the rates of major adverse events throughout the 30-day follow-up period (1.8 and 2.7%, respectively).

Finally, a recent sub-analysis of the TRILUMINATE Pivotal study [68] also reported the outcomes of patients with CIEDs, including a total of 98 patients. Individuals with a CIED were older (80.2 ± 8.6 vs. 78.2 ± 7.6 years; *p* = 0.02) and had higher rates of renal dysfunction (46.9% vs. 31.5%; *p* = 0.004) at the baseline, while lead-related TR was found in 15.5% of cases. In regard to procedural outcomes, procedural times were significantly shorter in those with CIED, potentially due to fewer clips implanted; however, the procedural duration was higher in patients with lead-related TR (149.5 ± 87.5 min). A TR grade of moderate or lower was achieved in similar rates in the two cohorts at both the 30-day (88% vs. 87%) and 1-year (81% vs. 84%) follow-ups. As in previous studies, significant improvements from the baseline were noted in NYHA class and KCCQ score (all *p* < 0.0001), with no difference in clinical endpoints and no lead dysfunction or removal during the 1-year follow-up.

The aforementioned studies provide early notions of the safety and efficacy of T-TEER in patients with both lead-associated and lead-related TR, showing comparable results to patients without leads. However, these results are limited due to the longer-term follow-up, the low number of studies, and the distinctive differences between lead-associated and lead-related TR, which have not been investigated in isolation. T-TEER is a promising alternative for patients at high risk of lead entrapment, which would result in significant adverse events. As noted in the previous studies, no interactions of the T-TEER devices with the lead were identified, with the exception of one study noting an alteration in the pacing thresholds. Future studies are necessary in order to identify whether distinct parameters related to the lead presence and its interaction with the valve could lead to suboptimal outcomes after T-TEER. Importantly, identifying which patients should undergo lead extraction or tricuspid intervention is crucial, and further data are needed in this context to support informed decision making.

## 7. T-TEER and Primary TR

Even though major trials have mostly included patients with secondary TR, the use of T-TEER could be employed as well in primary TR. The frequency of primary TR is much lower than that of secondary TR, with a prevalence estimated at 8–10% [69]. Primary TR is the result of abnormalities of any part of the tricuspid valve, namely the leaflets, chordae, papillary muscles, or annulus, due to either congenital or acquired causes, with the most common being tricuspid leaflet degeneration [26]. From the total number of patients undergoing T-TEER, 10–20% have primary TR [16,17,70]. Even though guidelines suggest surgical intervention as the optimal treatment [71], as surgical interventions have high mortality rates, especially in patients with comorbidities and RV dysfunction, T-TEER could serve as an alternative, similar to secondary TR, for the resolution of the pathology. However, in contrast to secondary TR, the inherent pathology of the leaflets leading to distinct leaflet anatomy among each patient could compromise optimal leaflet coaptation and therefore the procedural success. To date, given the low rates of primary TR in major trials and the absence of reports of specific data for these patients, limited evidence exists regarding the safety and efficacy of T-TEER for this phenotype (Table 5).

Dannenberg et al. [72] provided one of the first evaluations of T-TEER feasibility and efficacy in primary TR. Specifically, they enrolled a total of 339 patients, out of which 44 (13%) had primary TR and 295 (87%) had secondary TR, who underwent T-TEER with both TriClip and PASCAL devices. A trend towards more female patients was noted in the primary TR cohort (66 vs. 52%; *p* = 0.08), while they had a significantly lower TRI-SCORE (7 ± 6 vs. 9 ± 10; *p* = 0.015). Moreover, patients with primary TR had a similar TR baseline severity, better RV function, and smaller left atrial volumes, indicating less remodeling of the cardiac chambers. A total of 66% of patients had prolapse and 36% a flail leaflet, while a defect in all tricuspid leaflets was noted in 36% of individuals. In regard to the procedural outcomes, T-TEER significantly reduced delta vena contracta width (9 ± 5 vs. 9 ± 6 mm, *p* = 0.96), delta TR grade (median 2 (2) vs. 2 (2), *p* = 0.94), as well as residual TR grade ≤ 2 (76 vs. 78%, *p* = 0.85) in both primary and secondary TR, with no safety concerns. These results remain significant after propensity matching. Thus, T-TEER was able to reduce post-procedural TR severity to a similar extent as secondary TR, without increased procedural complications.

One-year data from the previous cohort are provided by Rudolph et al. [73], who report the follow-up of 201 patients undergoing T-TEER (13.4% primary TR and 86.6% secondary TR). At the time of the follow-up, the rate of moderate or lower levels of residual TR was 80.0% in patients with primary TR (*p* = 0.66 from the post-procedural evaluation) and 61.8% in patients with secondary TR (*p* = 0.12 from the post-procedural evaluation). Safety events were comparable between groups, with single leaflet detachment occurring in 7.4 and 6.3%, respectively (*p* = 0.69). Along with a reduction in TR grade at 1 year, both arms had significant improvements in NYHA class, with 75.0% of the primary TR and 65.7% of the secondary TR cohort being classified as NYHA class II or lower (*p* < 0.001 from the baseline for both groups). Finally, even though more patients with secondary TR experienced HF hospitalization during the follow-up, the all-cause mortality was similar between the two groups (25.9 vs. 24.7%; *p* > 0.99).

Further data on patients with primary TR have also been recently published by the Primary TR Registry [74], which included 114 patients undergoing T-TEER with both available devices. Overall, the patients included in the study were old (with a mean age of 79.9 years) and mostly male (53.5%), with NYHA class III or IV (83.3%) and a median TRI-SCORE of 5.0. Most patients had leaflet prolapse (61.4%), followed by flail leaflet (28.1%) and restricted leaflet (10.5%), while no cases of perforation were included. Regarding procedural outcomes, successful device deployment was achieved in 95.6% of cases, with an immediate reduction in TR to moderate or lower levels in 83.3% of patients. Importantly, the in-hospital mortality was low (1.8%), with a rate of single leaflet detachment of 3.5%. At the 1-year follow-up, the TR reduction to moderate or lower levels was sustained in 79.7% of individuals, with a parallel significant improvement in NYHA class (66.5% class I or II; *p* < 0.001 from the baseline) and echocardiographic signs of reverse right heart remodeling. Notably, the estimated 1-year mortality was 17.3% (95% CI: 10.0–29.0%) and the estimated HF hospitalization rate was 11.1% (95% CI: 3.7–17.9%), which are comparable to real-world data, but higher than the data from early T-TEER studies. Finally, when the authors integrated a surgical cohort in the analysis, they showed a more pronounced TR reduction with surgery (moderate or lower levels of TR at 83.3 vs. 97.3%; *p* < 0.001) and a trend for better in-hospital mortality with T-TEER (1.8% vs. 6.3%; *p* = 0.062), without any significant difference in 1-year mortality rates (17.3% vs. 9.0%; *p* = 0.25).

The available studies to date show early signs of the safety and efficacy of T-TEER in primary TR, which could serve as an alternative to surgical interventions for select patients. However, considering the small sample size, limited available studies, and the absence of extensive long-term follow-ups, further analyses are needed to validate these findings in comparison to the surgical gold standard. Importantly, as primary TR may present with a diverse leaflet anatomy compromising procedural success, it is important to identify scores of anatomic suitability, as in secondary TR. Of note, Sugiura et al. [74] note that, even though their results were comparable to surgery, they should be interpreted while keeping in mind that individuals undergoing surgery may not be anatomically suitable for T-TEER. Also, given that no patients with leaflet perforation were included and that the TR reduction with T-TEER was suboptimal in patients with restricted leaflet anatomy, it is necessary to evaluate the safety and efficacy of T-TEER among the different primary TR leaflet morphologies in order to establish the indications for this procedure and identify potential benefits in comparison to surgery.

## 8. Future Directions

**Figure 1:**
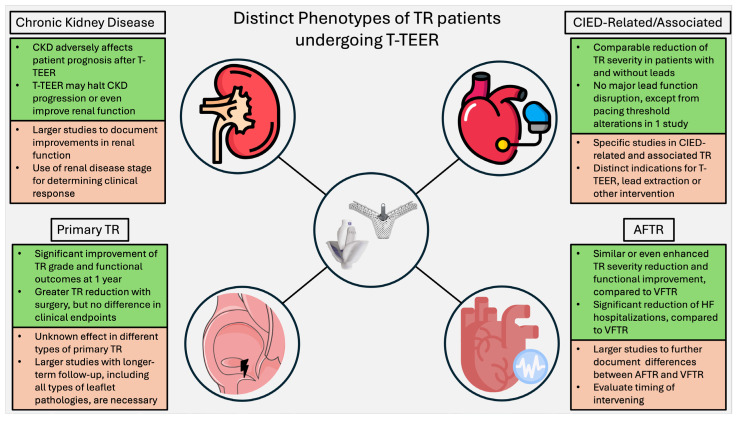
Overview of distinct patient phenotypes undergoing T-TEER, highlighting available evidence for each phenotype and future research directions/gaps in knowledge. *Abbreviations: AFTR: atrial functional tricuspid regurgitation; CIED: cardiac implantable electronic device; CKD: chronic kidney disease; T-TEER: tricuspid transcatheter edge-to-edge repair; TR: tricuspid regurgitation; VFTR: ventricular functional tricuspid regurgitation*.

TR includes a diverse combination of different anatomies and patient phenotypes that could affect patient management; therefore, emerging interventions require careful selection and should be well defined. Along with the proven safety and efficacy of T-TEER in major randomized trials evaluating mostly patients with secondary TR, analyses of the available data reveal that this procedure could be safely and efficiently performed in several subsets of patients (Figure 1). Therefore, beyond established anatomic criteria, the further stratification of patients based on the potential clinical benefit of each phenotype may be useful in clinical practice, even though more evidence from larger studies is needed to address certain considerations for such patients (Table 6). Interestingly, despite this diversity in presentation and characteristics, which are specific for each cohort, these differences do not seem to importantly diminish the benefit associated with T-TEER or increase safety events. Identifying whether this intervention has any particular benefit in such subsets of patients is important in order to further clarify patient selection criteria and identify the optimal timing for intervention. Importantly, as almost no trial, to date, has shown the benefit of tricuspid interventions over conservative therapy in terms of survival in their overall cohort, analyzing subsets of this pathology based on anatomic, pathophysiologic characteristics and comorbidities could provide significant prognostic information. Notably, the only study that has shown a reduction in mortality events with the use of T-TEER over conservative treatment was a recent investigation by Schlotter et al. [75], which stratified patients based on a risk score including baseline and echocardiographic characteristics into early, intermediate, and advanced disease. In this study, only patients in the intermediate arm had significantly decreased mortality rates after T-TEER, compared to conservative treatment, showcasing that the careful and timely selection of patients can result in substantial clinical benefit. Further investigations, focusing on predictors of enhanced outcomes as well as differences in the treatment of different patient phenotypes, will inform patient selection for T-TEER and assist the early identification of patients that would benefit the most, in terms of improvements in symptoms and clinical outcomes, after the intervention.

Even though T-TEER is the most studied and used transcatheter intervention for TR, there are several other procedures that could also be suitable for some patients, including transcatheter tricuspid valve annuloplasty, transcatheter tricuspid valve replacement (TTVR), and bicaval valve implantation [76]. Transcatheter annuloplasty aims to repair TR by correcting annular dilatation, which is often present in patients with secondary TR. Several studies have highlighted the safety and efficacy of transcatheter tricuspid annuloplasty, with Nickenig et al. [77] showing a significant reduction in TR (72% of patients with ≤ moderate TR; *p* = 0.016 from the baseline) and an improvement of functional status (82% of patients NYHA class I/II; *p* = 0.002 from the baseline) at the 2-year follow-up. These results have also been replicated by subsequent studies evaluating both the Cardioband and the novel K-Clip system [78,79]. Notably, as already mentioned, the safety and feasibility of annuloplasty have recently been proven in both patients with AFTR [59] and in the presence of leads [80]. Even though more studies are needed comparing the outcomes of T-TEER and annuloplasty in such settings, this procedure could provide a promising T-TEER alternative, especially in patient phenotypes in which annular dilation is the key pathophysiologic hallmark or the interaction of the device with the leaflets is unwanted. TTVR represents another option for TR interventions, which aims to eliminate TR not by valve repair, but by valve replacement. This alternative could be particularly beneficial for patients in whom significant residual TR is expected with T-TEER, therefore necessitating the use of a device that could provide sufficient TR elimination and result in enhanced patient outcomes. The TRISCEND II randomized study is the largest to report on this, comparing the Evoque valve to a valve with medical treatment [81]. Specifically, at 1 year, the win ratio favoring valve replacement was 2.02 (95% CI: 1.56 to 2.62; *p* < 0.001). The study showed a 99.1% reduction of TR to moderate or lower levels in the TTVR cohort, as compared to 16.1% in the control group, along with substantial improvements in NYHA class and exercise capacity. However, the Kaplan–Meier estimates for all-cause mortality at 1 year were 12.6 ± 2.1% for the TTVR group and 15.2 ± 3.3% for the control group, while for HF hospitalization, they were 20.9 ± 2.6% and 26.1 ± 4.1%, respectively, showing no significant difference between the groups. Importantly, significantly increased severe bleeding events (15.4 vs. 5.3%) and new pacemaker implantations (17.4 vs. 2.3%) were noted in the TTVR cohort, with no difference in other safety parameters. Positive results, in regard to echocardiographic TR grade and functional improvement, have also been reported with the use of the LuX-Valve system [82], with further studies awaited to investigate the long-term safety and efficacy of these valves. Finally, bicaval valves are another option included in TR interventions and consist of two bioprosthetic valves implanted in the superior and inferior vena cava in order to alleviate systemic congestion and extracardiac TR manifestations. Even though this procedure has more limited clinical applicability, especially when used for advanced and end-stage disease [26], it has been associated with significant functional and biochemical improvements as well as a reduction in organ dysfunction in this carefully selected cohort [83].

As greater experience is available in the field of transcatheter tricuspid valve therapies and as other interventions are becoming commercially available, such as the Evoque valve, an increase in interventions is expected, and, therefore, optimal patient selection for each alternative is of key importance. In this context, recently published algorithms aid patient selection based, mostly, on anatomic criteria, considering the lack of discrete patient phenotypes that could benefit more from each of the aforementioned procedures [27]. However, future studies should further investigate which is the optimal phenotype for T-TEER or other tricuspid interventions, based on additional parameters and distinct patient and disease characteristics, along with differences in the specific systems that are under investigation. It is certain that, even though the early evidence provided for the treatment of secondary TR with TTVR is promising, as in T-TEER, specific studies assessing the safety and efficacy of TTVR in patients with AFTR, CIED, and primary TR will be needed, as they represent distinct clinical entities with potentially different clinical outcomes that could alter patient management.

## 9. Conclusions

TR, along with anatomical challenges, represents a spectrum of different patient phenotypes with distinctive pathophysiologies and prognoses. The available studies for several TR phenotypes validate the safety and efficacy of T-TEER in reducing TR grade and improving symptom and functional status; however, limited data exist regarding significant improvements in clinical endpoints. Identifying predictors of enhanced clinical outcomes, as well as the optimal timing for tricuspid interventions, is key for the further implementation of such procedures in everyday practice and the identification of potential benefits in terms of mortality and adverse events.

## Figures and Tables

**Table 1:** Major single-arm, observational studies assessing the safety and efficacy of T-TEER at 1 year follow-up.

Study	Study Design	Patients (n)	Device Included	TR Less than Moderate or 2+	NYHA Class I/II	KCCQ Improvement	6MWT Distance Improvement
TRILUMINATE single-arm [15]	Observational, single-arm	85	TriClip	71% (vs. 8% at baseline, *p* < 0.0001)	83% (vs. 31% at baseline, *p* < 0.0001)	20 ± 2.61 points (*p* < 0.0001 from baseline)	303.2 ± 15.6 m (vs. 272.3 ± 15.6 m at baseline, *p* = 0.0023)
bRIGHT [16]	Observational, registry analysis	511	TriClip	81% (vs. 2% at baseline, *p* < 0.001)	75% (vs. 21% at baseline, *p* < 0.0001)	19 ± 26 points (*p* < 0.0001 from baseline)	-
PASTE [17]	Observational, registry analysis	1059	Pascal	83% (vs. 4% at baseline, *p* < 0.001)	66% (vs. 17% at baseline, *p* < 0.001)	-	40 ± 86 m (*p* < 0.001 from baseline)
CLASP TR EFS [18]	Observational, single-arm	65	Pascal	86% (vs. 3% at baseline, *p* < 0.001)	92% (vs. 29.2% at baseline, *p* < 0.001)	18 points (*p* < 0.001 from baseline)	311 ± 218 m (vs. 208 ± 107 m at baseline, *p* = 0.014)

Abbreviations: 6MWT: six-minute walking test; EFS: early feasibility study; KCCQ: Kansas City Cardiomyopathy Questionnaire; NYHA: New York Heart Association; TR: tricuspid regurgitation.

**Table 2:** Studies evaluating the effect of T-TEER on renal function.

Study	Year	Number of Participants	Intervention	Device Used	CKD Definition	Follow-Up	Improvement in eGFR	Predictors of Renal Improvement	Clinical Outcomes
Karam et al. [45]	2019	126	T-TEER (n = 59); T-TEER and concomitant M-TEER (n = 67)	MitraClip	Moderate to severe CKD: eGFR < 60 mL/min/1.73 m^2^	6 months	No change in eGFR in the overall cohort (50.7 ± 21.8 vs. 49.8 ± 21.9 mL/min/1.73 m^2^; *p* = 0.45); No change in eGFR in patients with moderate-to-severe CKD (37.5 ± 11.9 vs. 40.1 ± 17.3 mL/min/1.73 m^2^; *p* = 0.39).	NR	NR
Jorde et al. [44]	2024	572	T-TEER and OMT (n = 285) versus OMT (n = 287)	TriClip	Mild to moderate: <60 mL/min/1.73 m^2^; Moderate to severe: <45 mL/min/1.73 m^2^	12 months	No difference in the change of eGFR (−0.10 ± 0.79 vs. −2.22 ± 0.82 mL/min/1.73 m^2^; *p* = 0.063) from baseline. In patients with moderate or lower levels of TR at discharge, eGFR significantly increased after T-TEER, compared to controls (+0.30 ± 0.85 vs. −2.27 ± 0.82 mL/min/1.73 m^2^; *p* = 0.03) Similar increase noted in patients with procedural success and baseline renal dysfunction (+3.55 ± 1.04 vs. +0.07 ± 1.10 mL/min/1.73 m^2^; *p* = 0.022)	Procedural success (TR ≤ moderate)	TEER patients with moderate to severe renal impairment had numerically lower HF hospitalization rates compared to control patients (28.9% vs. 36.2%; *p* = 0.40)
Felbel et al. [46]	2024	92	T-TEER	TriClip	NR	3, 12 months	eGFR improvement (eGFR at follow-up greater than baseline) was present in 57.6% of patients	TR vena contracta reduction (OR: 1.35; 95% CI: 1.12–1.64 per mm; *p* = 0.002); Reduced pre-interventional TAPSE (OR 0.89; 95% CI: 0.79–0.9 per mm; *p* = 0.033)	An eGFR improvement greater than >9 mL/min was related with significantly decreased 1-year HF hospitalization rates (aHR: 0.22; 95% CI: 0.07–0.62; *p* = 0.005).

Abbreviations: aHR: adjusted hazard ratio; CI: confidence interval; eGFR: estimated glomerular filtration rate; HR: hazard ratio; HF: heart failure; NR: not reported; M-TEER: mitral transcatheter edge-to-edge repair; OMT: optimal medical therapy; OR: odds ratio; T-TEER: tricuspid transcatheter edge-to-edge repair; TAPSE: tricuspid annular plane systolic excursion; TR: tricuspid regurgitation.

**Table 3:** Studies evaluating the effect of T-TEER in patients with AFTR.

Study	Year	Number of Participants	Intervention	Device Used	AFTR (%)	Follow-Up	Improvement in TR Grade (Moderate or Less)	Improvement in Functional Outcomes (NYHA I/II))	Improvement in Clinical Outcomes
Schlotter et al. [52]	2022	651	T-TEER (n = 213) or OMT (n = 418)	MitraClip and Pascal	14.8%	12 months	86 vs. 81%; *p* < 0.001 from baseline for both arms	75 vs. 60%; *p* < 0.001 from baseline for both arms	AFTR was associated with a decreased rate of mortality and HF hospitalizations at 1 year (12% vs. 36%; *p* = 0.017) This association remained independent of NYHA class, RV function, sex, baseline comorbidities, and baseline TR grade
Scheffler et al. [56]	2025	136	T-TEER	TriClip and Pascal	19.9	12 months	94.5 vs. 96.3%, *p* = 1.0	NR	Significantly increased rates of all-cause mortality in patients with VFTR (11.1 vs. 32.1%, *p* = 0.01); Significantly increased rates of HF hospitalization in patients with VFTR (7.4 vs. 29.4%, *p* = 0.02). In multivariate analysis, AFTR was independently associated with lower rates of the composite of all-cause mortality and HF hospitalization (HR: 0.21, 95% CI: 0.06–0.7, *p* < 0.01)
Russo et al. [57]	2023	298	T-TEER	MitraClip/TriClip and Pascal	22	10 months	77 vs. 85%; *p* = 0.001 from baseline for both	NR	AFTR was significantly associated with increased survival rates at follow-up (91% vs. 72%, *p* = 0.02); In a multivariate model, VFTR was associated with a non-significant trend towards increased mortality (HR 2.11, 95% CI 0.99–4.47, *p* = 0.051, respectively)
Stolz et al. [58]	2024	641	T-TEER	MitraClip/TriClip and Pascal	31	12 and 24 months	86.9 vs. 80.4%; *p* = 0.005 at 12 months	62 vs. 54%; *p* = 0.033 at 12 months	At 24 months, AFTR was associated with increased survival and HF hospitalization-free rates (66.3% vs. 47.5%; *p* < 0.001). AFTR was an independent predictor of freedom from HF hospitalization (HR: 0.65; 95% CI: 0.45–0.96; *p* = 0.025)

Abbreviations: AFTR: atrial functional tricuspid regurgitation; HR: hazard ratio; HF: heart failure; NR: not reported; NYHA: New York Heart Association; OMT: optimal medical therapy; RV: right ventricle; T-TEER: tricuspid transcatheter edge-to-edge repair; TR: tricuspid regurgitation; VFTR: ventricular functional tricuspid regurgitation.

**Table 4:** Studies evaluating the effect of T-TEER in patients with transvalvular leads/CIEDs.

Study	Year	Number of Participants	Intervention	Device Used	Patients with CIED (%)	Follow-Up	Improvement in TR Grade (Moderate or Lower Levels)	Improvement in Functional Outcomes (NYHA I/II))	Improvement in Clinical Outcomes
Braun et al. [64]	2017	41	T-TEER	NR	31.7	1 month	85 vs. 71%; *p* < 0.001 and =0.02 from baseline, respectively	46 vs. 64%; *p* = 0.01 and <0.001 from baseline, respectively	NR
Lurz et al. [65]	2019	102	T-TEER with or without concomitant M-TEER (in 64 and 48% of patients, respectively)	NR	32.4	1 month	TR grade NR; No significant difference in EROA reduction (0.29 ± 0.25 vs. −0.23 ± 0.34 cm^2^; *p* = 0.86)	NR	NR
Taramasso et al. [66]	2020	470	TV interventions, T-TEER in 79.2% of patients; Concomitant M-TEER in 33%	MitraClip, Pascal, FORMA, Cardioband, TriCinch, Trialign, CAVI, NaviGate	25.7	1, 12 months	73 vs. 70%; *p* = 0.60 and *p* < 0.05 from baseline for both	65 vs. 66%; *p* = 0.30 and *p* < 0.05 from baseline for both	Survival was similar in the overall group among arms at 12 months (73.6 ± 5.2 vs. 80.7 ± 3.1%; *p* = 0.30). Similar results were reported in those with isolated TV intervention (82.0 ± 8.4 vs. 82.9 ± 4.2%; *p* = 0.70)
Goebel et al. [67]	2025	511	T-TEER	TriClip	21.5	1 month	71 vs. 78%; *p* < 0.0001 for both from baseline	75 vs. 80%; *p* < 0.0001 for both from baseline	Cardiovascular mortality was 0% for subjects with leads and 1% for subjects without leads. Major adverse event rate was similar among groups (1.8 and 2.7%)
Naik et al. [68]	2025	469	T-TEER	TriClip	20.9	12 months	81 vs. 84%; *p* < 0.0001 for both from baseline	77 vs. 85%; *p* < 0.0001 for both from baseline	No difference in all-cause mortality (11.2 vs. 10.8%; *p* = 0.90), cardiovascular death (8.2 vs. 7.5%; *p* = 0.84), and HF hospitalizations (16.3 vs. 15.9%; *p* = 0.92) among groups

Abbreviations: CIED: cardiac implantable electronic device; HF: heart failure; M-TEER: mitral transcatheter edge-to-edge repair; NR: not reported; NYHA: New York Heart Association; T-TEER: tricuspid transcatheter edge-to-edge repair; TR: tricuspid regurgitation; TV: tricuspid valve.

**Table 5:** Studies evaluating T-TEER in patients with primary MR.

Study	Year	Number of Participants	Intervention	Device Used	Primary TR (%)	Follow-Up	Improvement in TR Grade (Moderate or Lower)	Improvement in Functional Outcomes (NYHA I/II))	Improvement in Clinical Outcomes
Dannenberg et al. [72]	2024	339	T-TEER	MitraClip/TriClip and Pascal	13	Post-procedural results	76 vs. 78%, *p* = 0.85	NR	NR
Rudolph et al. [73]	2025	201	T-TEER	NR	13.4	12 months	80 vs. 61.8%; *p* = 0.66 and 0.12 from post-procedural results, respectively	75 vs. 65.7%; *p* < 0.001 from baseline for both	All-cause mortality was not different between groups (25.9 vs. 24.7%; *p* > 0.99). There was a trend towards lower HF hospitalization rates in patients with primary TR (3.7 vs. 22.4%; *p* = 0.08).
Sugiura et al. [74]	2025	114	T-TEER	MitraClip/TriClip and Pascal	100	12 months	79.7%; *p* < 0.001 from baseline	66.5%; *p* < 0.001 from baseline	In-hospital mortality: 1.8%; Estimated 1-year all-cause mortality: 17.3% (95% CI: 10.0–29.0%); Estimated HF hospitalization event rate: 11.1% (95% CI: 3.7–17.9%)

Abbreviations: CI: confidence interval; NR: not reported; HF: heart failure; NYHA: New York Heart Association; T-TEER: tricuspid transcatheter edge-to-edge repair; TR: tricuspid regurgitation.

**Table 6:** Overview of established anatomic criteria of T-TEER suitability, as well as novel phenotypes and their considerations.

Established Anatomical Criteria of T-TEER Optimal Phenotype	
Small coaptation gap (≤7 mm)	
Septolateral gap	
Anteroseptal or central jet location	
Trileaflet valve morphology	
Confined prolapse or flail	
Sustained RV function	
Low RA volume	
**Phenotype-based Patient Selection**	
**Patient Phenotype**	**Considerations**
Renal dysfunction	Potential survival benefit in patients with moderate CKD Potential improvement of renal function
AFTR	Enhanced clinical outcomes compared to VFTR, either phenotype-associated or disease stage-associated
Primary TR	Potential suboptimal results in restricted leaflet anatomy
Transvalvular leads	Despite their feasibility and absence of lead dysfunction, more data are needed to establish their safety in lead-related and lead-associated TR

Abbreviations: AFTR: atrial functional tricuspid regurgitation; CKD: chronic kidney disease; RA: right atrium; RV: right ventricle; T-TEER: tricuspid transcatheter edge-to-edge repair; TR: tricuspid regurgitation.

## Data Availability

No new data were created, as this is a review article.
